# Arginine deiminase augments the chemosensitivity of argininosuccinate synthetase-deficient pancreatic cancer cells to gemcitabine via inhibition of NF-κB signaling

**DOI:** 10.1186/1471-2407-14-686

**Published:** 2014-09-20

**Authors:** Jiangbo Liu, Jiguang Ma, Zheng Wu, Wei Li, Dong Zhang, Liang Han, Fengfei Wang, Katie M Reindl, Erxi Wu, Qingyong Ma

**Affiliations:** Department of Hepatobiliary Surgery, First Affiliated Hospital, Medical college of Xi’an Jiaotong University, 277 West Yanta Road, Xi’an, Shaanxi 710061 China; Department of General Surgery, First Affiliated Hospital/Cancer Institute, Henan University of Science and Technology, 24 Jinghua Road, Luoyang, 471000 China; Department of Oncology, First Affiliated Hospital, Xi’an Jiaotong University, 76 West Yanta Road, 710061 Xi’an, China; Department of Pharmaceutical Sciences, North Dakota State University, 58105 Fargo, ND USA; Department of Biological Sciences, North Dakota State University, 58105 Fargo, ND USA

## Abstract

**Background:**

Pancreatic cancer is a leading cause of cancer-related deaths in the world with a 5-year survival rate of less than 6%. Currently, there is no successful therapeutic strategy for advanced pancreatic cancer, and new effective strategies are urgently needed. Recently, an arginine deprivation agent, arginine deiminase, was found to inhibit the growth of some tumor cells (i.e., hepatocellular carcinoma, melanoma, and lung cancer) deficient in argininosuccinate synthetase (ASS), an enzyme used to synthesize arginine. The purpose of this study was to evaluate the therapeutic efficacy of arginine deiminase in combination with gemcitabine, the first line chemotherapeutic drug for patients with pancreatic cancer, and to identify the mechanisms associated with its anticancer effects.

**Methods:**

In this study, we first analyzed the expression levels of ASS in pancreatic cancer cell lines and tumor tissues using immunohistochemistry and RT-PCR. We further tested the effects of the combination regimen of arginine deiminase with gemcitabine on pancreatic cancer cell lines *in vitro* and *in vivo*.

**Results:**

Clinical investigation showed that pancreatic cancers with reduced ASS expression were associated with higher survivin expression and more lymph node metastasis and local invasion. Treatment of ASS-deficient PANC-1 cells with arginine deiminase decreased their proliferation in a dose- and time-dependent manner. Furthermore, arginine deiminase potentiated the antitumor effects of gemcitabine on PANC-1 cells via multiple mechanisms including induction of cell cycle arrest in the S phase, upregulation of the expression of caspase-3 and 9, and inhibition of activation of the NF-κB survival pathway by blocking NF-κB p65 signaling via suppressing the nuclear translocation and phosphorylation (serine 536) of NF-κB p65 *in vitro*. Moreover, arginine deiminase can enhance antitumor activity of gemcitabine-based chemotherapy in the mouse xenograft model.

**Conclusions:**

Our results suggest that arginine deprivation by arginine deiminase, in combination with gemcitabine, may offer a novel effective treatment strategy for patients with pancreatic cancer and potentially improve the outcome of patients with pancreatic cancer.

**Electronic supplementary material:**

The online version of this article (doi:10.1186/1471-2407-14-686) contains supplementary material, which is available to authorized users.

## Background

Pancreatic cancer is the fourth most common cause of cancer-related deaths in western countries, with a median overall survival of less than 6 months and a 5-year survival rate of less than 6% [1, 2]. In 2014, it is estimated that 46,420 Americans will be newly diagnosed with pancreatic cancer and 39,590 will die of the disease [2]. Because of aggressive growth, early local invasion, and tumor metastasis, the majority of patients (over 80%) are diagnosed at an unresectable stage [3]. Gemcitabine (2'-Deoxy-2', 2'-difluorocytidine; GEM)-based chemotherapy has been used as a palliative cancer treatment for more than two decades and is currently the first-line chemotherapeutic agent for treatment of patients with advanced pancreatic cancer. However, given that pancreatic cancer is highly resistant to chemotherapeutic agents, a number of clinical trials show that GEM alone or in combination with other regimens such as cetuximab, or S-1 [an oral fluorourail (FU) derivative], does not improve the overall survival of pancreatic cancer patients [4–6]. Therefore, it is imperative to develop novel therapeutic strategies.

Arginine can be synthesized from citrulline by the enzymes of the urea cycle, namely argininosuccinate synthetase (ASS) and argininosuccinate lyase (ASL), and is, therefore, regarded as a nonessential amino acid for humans and mice [7]. Some human cancers, such as melanoma, lung cancer, renal cell carcinomas, and hepatocellular carcinomas [8–10] do not express ASS and are highly sensitive to arginine deprivation via arginine deiminase (ADI). ADI is an arginine deprivation agent capable of degrading arginine into citrulline [11, 12]. ADI eliminates intracellular arginine by reducing the extracellular and plasma levels, thereby producing an arginine shortage in the ASS-deficient tumor cells, but not affecting cells that express ASS [10, 13, 14]. A recent study demonstrated that several pancreatic cancer cells exhibit reduced ASS expression, and the growth of these cells *in vitro* and *in vivo* is inhibited via arginine elimination using a polyethylene glycol-modified ADI (PEG-ADI) [15].

GEM, a pyrimidine-based antimetabolite, has been used for the treatment of pancreatic cancer for two decades [16, 17]. It has been demonstrated that GEM activates the S-phase checkpoint via inhibition of DNA replication [18]. As documented above, pancreatic cancers are often resistant to GEM through several molecular mechanisms [19–24]. NF-κB plays a critical role in activating transcriptional events that lead to cell survival, and activation of this signaling pathway is associated with GEM chemoresistance in pancreatic cancer cells [23, 25, 26]. Agents that block NF-κB activation could reduce chemoresistance to GEM and may be used in combination with GEM as a novel therapeutic regimen for treating pancreatic cancer [27–30]. Previous research has demonstrated that arginine deprivation therapy and the associated agent ADI may be a promising therapy for pancreatic cancer [15]. However, whether ADI potentiates the anticancer activities of GEM in pancreatic cancer cells and its precise mechanisms are not clear.

In this study, we aimed to examine the effects and mechanisms of ADI alone and in combination with GEM on the survival of pancreatic cancer cells *in vitro* and *in vivo* in order to develop a novel effective therapeutic strategy for treating pancreatic cancer. Our results show that pancreatic cancer cells lacking ASS expression have high sensitivity to arginine deprivation by ADI. Further, when ADI was combined with GEM in ASS-negative pancreatic cancer cells, NF-κB signaling was suppressed and more cell death was induced *in vitro* and *in vivo*. Clinically, pancreatic cancer patients with reduced ASS expression may have shorter survival times.

## Methods

### Reagents and chemicals

Diamidino-2-phenylindole (DAPI), crystal violet, Dimethyl Sulfoxide (DMSO), methyl thiazolyl tetrazolium (MTT), propidium iodide (PI), and RNase were obtained from Sigma Chemical (St. Louis, MO, USA). Bicinchoninic acid (BCA) protein assay reagent was from Pierce Chemical (Rockford, IL, USA). The ADI gene was cloned from the *M. arginini* genomic DNA, and the 46 kDa ADI recombinant protein (Additional file 1: Figure S1) was produced as previously described [31]. ADI activity was determined by measuring the formation of L-citrulline from L-arginine following a modified method using diacetyl monoxime thiosemicarbazide [32]. One unit of ADI activity is defined as the amount of enzyme catalyzing 1 μmol of L-arginine to 1 μmol of L-citrulline per min under the assay conditions. Finally, the measured activity of the ADI was 30 U per mg protein. GEM was purchased from Eli Lilly France SA (Fergersheim, France).

### Cell lines and cell culture

Human primary pancreatic cancer cell lines MIA PaCa-2, PANC-1, and BxPC-3, and spleen metastatic pancreatic cancer cell line SW1990, breast cancer cell lines MDA-MB-453, BT474, MDA-MB-231, and MCF-7, and hepatocellular carcinoma (HCC) cell lines HepG2 and MHCC97-H were all purchased from the American Type Culture Collection (ATCC). All cell lines were maintained in the recommended medium (HyClone, Logan, USA) containing 10% heat-inactivated fetal bovine serum (HyClone) and 1% penicillin/streptomycin (HyClone) in a humidified (37°C, 5% CO_2_) incubator. Plastic wares for cell culture were obtained from BD Bioscience (Franklin Lakes, NJ).

### Tissue samples and immunohistochemistry

Thirty-seven paraffin-embedded pancreatic cancer tissues were obtained from the First Affiliated Hospital of Medical College, Xi’an Jiaotong University, between 2007 and 2010. The paraffin-embedded tissue samples were then sliced into consecutive 4-μm-thick sections and prepared for immunohistochemical (IHC) studies. IHC staining was performed using an ultrasensitive SP-IHC kit (Beijing Zhongshan Biotechnology, Beijing, China), according to the manufacturer’s protocol. Briefly, after dewaxing and rehydration, the antigen was heat-retrieved, endogenous peroxidase was quenched, and the sample was blocked with 10% BSA for 30 min at room temperature. The slides were then immersed in either primary anti-ASS1 (H231; Santa Cruz Biotechnology, Santa Cruz, CA, USA) or anti-survivin (N111; Bioworld, Minneapolis, USA) rabbit polyclonal antibodies overnight at 4°C in a humid chamber, followed by rinsing and incubating with the goat anti-rabbit secondary antibody kit. The slides were stained with the 3,3-diaminobenzidine tetrahydrochloride (DAB) kit (Beijing Zhongshan Biotechnology, Beijing, China) and were subsequently counterstained with hematoxylin. Two pathologists assessed the IHC results as described previously [33]. Finally, the images were examined under a light microscope (Olympus, Tokyo, Japan). The Ethical Review Board Committee of the First Affiliated Hospital of Medical College, Xi’an Jiaotong University, China, approved the experimental protocols and informed consent was obtained from each patient who contributed tissue samples.

### Reverse transcription-polymerase chain reaction (RT-PCR) and quantitative-real time RT-PCR

Total RNA from cells was prepared using trizol (Invitrogen, Carlsbad, CA, USA) according to the manufacturer’s protocol [34]. Subsequently, the total RNA was reverse-transcribed into cDNA using a Takara Reverse Transcription Kit (Takara, Dalian, China) according to the manufacturer’s recommendations. Reverse transcription-polymerase chain reaction (RT-PCR) was performed as previously described [35]. For quantitative-real time (qRT)-PCR reactions, 2 μL of cDNA was mixed with a reaction mix containing 10 μL of SYBR Green (Takara), 0.8 μL of primers, and water for a total reaction volume of 20 μL. For detecting of ASS1, caspase-3, caspase-9, Bax, Bcl-2, and survivin at mRNA levels, the following gene specific primers (Beijing Dingguo Changsheng Biotechnology) were designed as follows:

- ASS1-sense: 5'-AGTTCAAAAAAGGGGTCCCT-3',
- ASS1-antisense: 5'-TTCTCCACGATGTCAATACG-3';
- Caspase-3-sense: 5'-GTAGAAGAGTTTCGTGAGTGC-3',
- Caspase-3-antisense: 5'-TGTCCAGGGATATTCCAGAG-3';
- Caspase-9-sense: 5'-GCCATGGACGAAGCGGATCGGCGG-3',
- Caspase-9-antisense: 5'-GGCCTGGATGAAGAAGAGCTTGGG-3';
- Survivin-sense: 5'-TCCACTGCCCCACTGAGAAC-3',
- Survivin-antisense: 5'-TGGCTCCCAGCCTTCCA-3';
- Bax-sense: 5'-GGCTGGACATTGGACTTC-3',
- Bax-antisense: 5'-AAGATGGTCACGGTCTGC-3';
- Bcl-2-sense: 5'-GTGTGGAGAGCGTCAACC-3',
- Bcl-2-antisense: 5'-CTTCAGAGACAGCCAGGAG-3';
- GAPDH-sense: 5'-CTCTGATTTGGTCGTATTGGG-3',
- GAPDH-antisense: 5'-TGGAAGATGGTGATGGGATT-3';

The number of specific transcripts detected was normalized to the level of GAPDH. Relative quantification of gene expression (relative amount of target RNA) was determined using the equation 2 ^(−ΔΔ Ct)^.

### Immunofluorescence

Cells were grown on glass coverslips, fixed with 4% paraformaldehyde for 10 min at room temperature, and then incubated with or without (control) the primary anti-ASS (H231) antibody overnight; the coverslips were then washed and incubated with the appropriate secondary antibody conjugated with FITC for 1 h at room temperature. DAPI was used to stain the nuclei. The coverslips were mounted onto slides, and the cells were viewed for evaluating ASS expression using a Leica TCS-SP2 confocal scanning microscope (Leica, Heidelberg, Germany).

### Western blot analysis

Total protein from pancreatic cancer tissues or cells was extracted following lysis in the RIPA lysis buffer (150 mM NaCl, 50 mM Tris, 1% NP-40, 0.25% sodium deoxycholate, and 1 mM EGTA) supplemented with the protease inhibitor cocktail (Sigma, St, Louis, USA) for 30 min [36]. The resulting debris was removed by centrifugation, and the supernatant containing the protein lysate was collected. For preparation of the nuclear extracts, cells in the control and experimental groups were treated for the indicated times, then incubated on ice for 30 min followed by preparation of the nuclear extracts using a nuclear extract kit (Pierce) according to the manufacturer's instructions. The cellular protein content was determined using the BCA kit (Beyotime Biotechnology, Nantong, China), and the cell lysates were separated on a 10% SDS-PAGE gel followed by electro-transfer onto a Millipore PVDF membrane (Billerica, USA). After being blocked with 5% non-fat milk in TBST, the membranes were incubated with the respective primary antibodies (pSTAT3 [Tyr705], total-STAT3, p-ERK1/2 [Thr202/Tyr204], and ERK1/2 [all from Cell Signaling Technology, Beverly, USA]; p-Akt [Thr308], total-Akt, total NF-κB p65, caspase-3, caspase-9, XIAP, c-Jun, p21, p53, β-actin [all from Santa Cruz Biotechnology]; survivin, p-c-Jun [S73], p-NF-κB p65 [S536], lamin B1 [all from Bioworld]; cyclin D1 [Boster, Wuhan, China], and ASS1 [Proteintech, Chicago, USA]) at 4 °C overnight, followed by 1:2000 horseradish peroxidase (HRP)-conjugated secondary antibodies (anti-mouse, anti-rabbit, anti-goat; Santa Cruz Biotechnology) for 2 h. Immunoreactive bands were visualized using an enhanced chemiluminescence kit (Millipore) and photographed by GeneBox analyzer (SynGene, UK). All analyses were performed in duplicate.

### Cell proliferation assay

Cell proliferation was determined by the MTT uptake method. Following an overnight culture in a 96-well plate in 200 μL of suitable medium, cells (5 × 10^3^/well) were treated with varying concentrations of ADI (0–10 mU/mL), GEM (0–10^5^ nM), or both agents for the indicated time. Then, MTT (5 mg/mL) was added and incubation was continued for 4 h, followed by termination of the reaction with 150 μL of DMSO per well. Absorbance values were determined at 490 nm on a Dias automatic microwell plate reader (Dynatech Laboratories, Chantilly, USA), using DMSO as the blank and cells cultured in untreated medium as the control group. The cell viability index was calculated using the formula of OD_sample_/OD_control_ × 100%, while inhibition ratio calculated by formula of (1 – OD_sample_/OD_control_) × 100%. Each experiment was repeated three times.

### Colony formation assay

Cells were seeded in a 6-well plate at a density of approximately 2.0 × 10^2^ per well (2 mL) and allowed to attach for 24 h. Next day, the adherent cells were treated with ADI (0, or 1.0 mU/mL) or GEM (0 or 100 nM), or both. When cells were treated with both ADI and GEM, the cells were first treated with ADI (0, or 1.0 mU/mL) for 12 h, followed by 100 nM GEM for another 12 h. After a total of 24 h of treatment, cells were cultured in DMEM and incubated under optimal culture conditions for 14 days, fixed with methanol, and stained with 0.1% crystal violet. Visible colonies were manually counted and photographed.

### Detection of cell apoptosis

Apoptosis was analyzed by three methods: 1) Flow cytometry: Apoptotic cells were analyzed using the Annexin-V-FITC/PI kit (BD, San Diego, USA) by a FACSCalibur flow cytometer (BD) according to the manufacturer's instructions. Briefly, cells (2 × 10^5^/well) were cultured in 6-well plates in the appropriate medium for 6 h prior to treatment with GEM (100 nM) and/or ADI (1 mU). Following incubation for the indicated times, cells were trypsinized and centrifuged, washed with PBS, and stained with Annexin V and PI in the dark. Samples were analyzed, and the percentage of apoptotic cells was evaluated. 2) *In situ* Annexin V/PI staining: Following the pretreatment as indicated in the flow cytometry, cells were washed with PBS and stained with 5 μL of anti-Annexin V-FITC and 5 μL of PI in 500 μL of binding buffer in the dark for 15 min and then examined using a fluorescence microscope. 3) Hoechst 33258/PI double staining: After treatment as indicated previously, cells were washed with PBS and stained with 0.1 mL of Hoechst 33258 (Beyotime Biotechnology, Nantong, China) and PI for 15 min. Stained cells were photographed under a fluorescence microscope.

### Cell cycle assay

Cells were harvested after treatment at different time points, and they were resuspended in PBS. The cells were then fixed in 2 mL of 70% ethanol and incubated on ice for 30 min, before being washed and treated with RNase A (100 μg/mL, 5 min) and stained with PI (50 μg/mL, 15 min). Cellular DNA content was analyzed in a Coulter Epics XL flow cytometer (Beckman-Coulter, Villepinte, France).

### NF-κB p65 nuclear translocation assay

After the drug treatment, the cells were incubated with the NF-κB p65 (C-20, Santa Cruz Biotechnology) antibody overnight. The subsequent processing was similar to the immunofluorescence assay. At the final step, the nuclear translocation of NF-κB p65 was viewed using a confocal scanning microscope (Leica).

### Tumorigenicity in a mouse xenograft model

Six- to eight-week-old male BALB/c athymic mice were kept under pathogen-free conditions according to institutional guidelines. Each aliquot of approximately 1.0 × 10^7^ PANC-1 pancreatic cancer cells suspended in 100 μL of PBS containing 20% of Growth Factor Reduced Matrigel (Becton Dickinson Labware, Flanklin, NJ, USA) was implanted subcutaneously into the mouse flank to establish xenograft tumors. After two weeks, the mice were randomly grouped into 4 groups with six animals in each group. Mice were intraperitoneally administered either PBS (vehicle), ADI (2 U/mouse), or GEM (100 mg/kg) alone or a combination of both ADI and GEM in 100 μL of PBS every four days. The tumor size was measured every three days and the tumor volume (in mm^3^) was calculated using the formula V = 0.4 × D × d^2^ (V, volume; D, longitudinal diameter; d, latitudinal diameter). Four weeks later, the mice were sacrificed and the tumors were excised and weighed. All animal experiments were conducted according to a protocol approved by the Institutional Animal Care and Use Committee of Xi’an Jiaotong University.

### Statistical analysis

Statistical analyses were performed using the SPSS software (version 16.0, SPSS Inc. Chicago, USA). Experimental data *in vitro* and *in vivo* were expressed as mean ± standard deviation (SD), and were analyzed by the Student’s unpaired t-test or one-way ANOVA. For frequency distributions, a χ^2^ test was used with modification by the Fisher’s exact test to account for frequency values less than 5. *P* < 0.05 was considered statistically significant.

## Results

### Expression of ASS in pancreatic cancer cells and tissue

**Figure 1:**
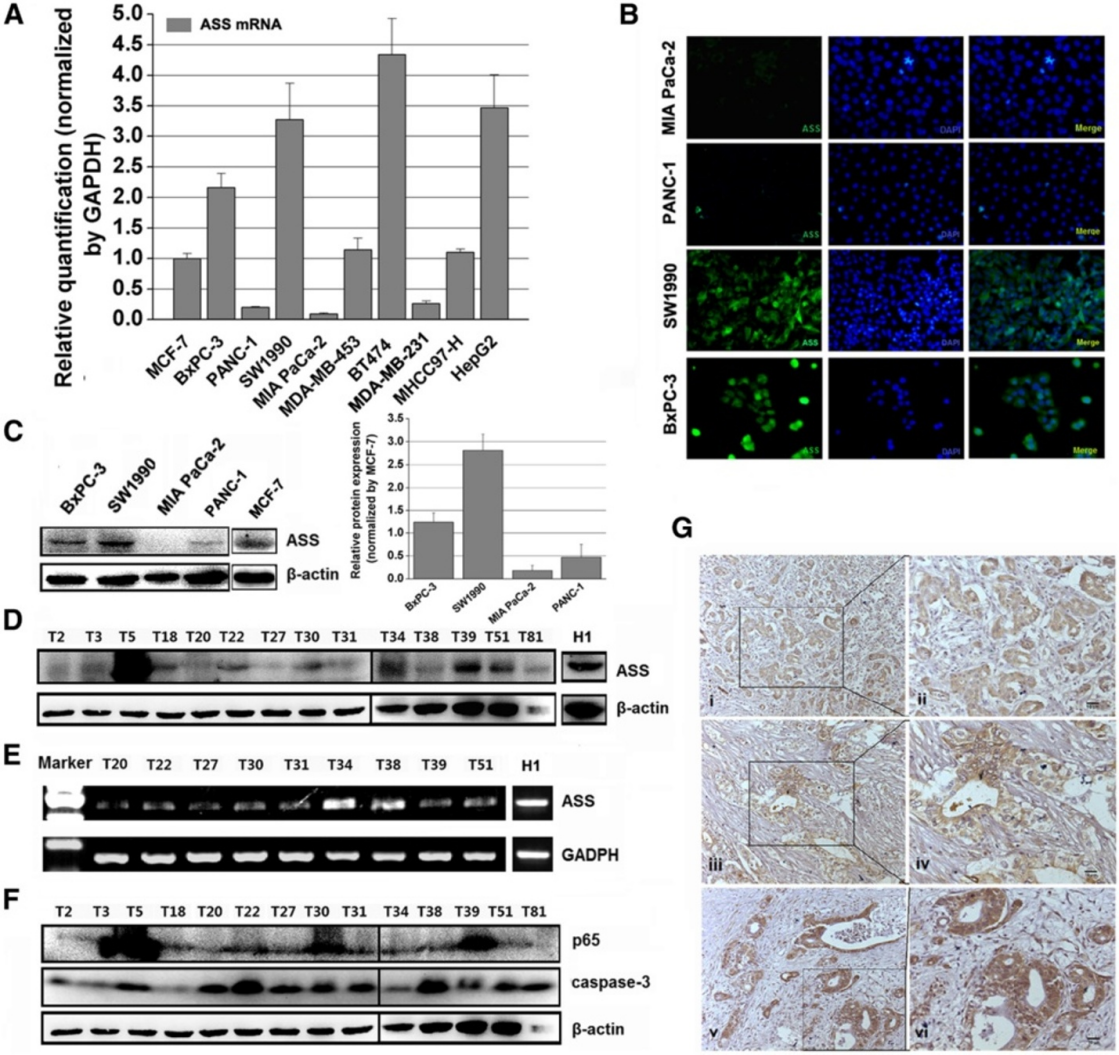
**The expression of ASS mRNA and protein in pancreatic cancer cell lines and human tissues. A**, The level of ASS mRNA in human pancreatic cancer cell lines MIA PaCa-2, PANC-1, BxPC-3, and SW1990, breast cancer (low ASS-deficient tumor [37]) cell lines MDA-MB-453, BT474, MDA-MB-231, and MCF-7, and hepatocellular carcinoma (high ASS-deficient tumor [37, 38]) cell lines HepG2 and MHCC97-H were examined by qRT-PCR assay. **B**, Cytoplasmic localization of ASS protein in MIA PaCa-2, PANC-1, SW1990, and BxPC-3 cells was verified by immunofluorescence assay. **C**, The expression level of ASS protein was evaluated by western blot assay in the pancreatic cancer cell lines. **D**, The expression of ASS protein in 14 pancreatic cancer tissues was detected by western blotting (H1 is a normal hepatic tissue obtained from a hepatorrhexis patient), yielding that the deficiency of ASS protein expression was up to 50% (7/14). **E**, Relative mRNA levels of ASS in 9 pancreatic cancer tissues were analyzed by RT-PCR (H1 as depicted Figure 1D), reporting similar ASS deficiency as protein level in examined specimens. **F**, The relative expression levels of the p65 subunit of NF-κB and caspase-3 proteins in 14 pancreatic cancer tissues were detected by western blotting. **G**, The expression of ASS or survivin was determined in pancreatic cancer tissue samples using immunohistochemistry (IHC). Images i and ii show ASS expression in a tissue sample obtained from a grade 3 pancreatic adenocarcinoma patient with chronic pancreatitis and without metastasis to the lymph nodes or other organs, while iii and iv show ASS expression in a tissue sample obtained from a grade 2 invasive adenocarcinoma characterized by lymphatic and liver metastases. Images in v and vi show survivin expression in a tissue sample from a grade 2 invasive adenocarcinoma with tumor extension and invasion into peripancreatic fat and multiple fibrous adhesions.

The mRNA expression levels of ASS, a key factor that determines sensitivity to arginine deprivation via ADI, were measured in several cancer cell lines, including pancreatic cancer, breast cancer (low ASS-deficient tumor [37]), and HCC (high ASS-deficient tumor [37, 38]) using qRT-PCR. The MCF-7 breast cancer cell line was used as a standard control for identifying ASS mRNA expression. The BxPC-3 (primary pancreatic cancer), SW1990 (spleen metastatic pancreatic cancer), BT474 (breast cancer), and HepG2 (HCC) cells expressed high levels of ASS mRNA and the PANC-1 (primary pancreatic cancer), MIA PaCa-2 (primary pancreatic cancer), and MDA-MB-231 (breast cancer) cells expressed low levels of ASS relative to MCF-7 cells (Figure 1A). The MDA-MB-453 (breast cancer) and MHCC97-H (HCC) cell lines expressed similar ASS mRNA levels as MCF-7 cells. Next, the expression level of ASS protein was evaluated by western blot assay in the four pancreatic cancer cell lines. The ASS protein expression pattern was similar to the ASS mRNA expression pattern in these cells (Figure 1C). Immunofluorescence analysis verified that ASS protein was located in the cytoplasm of BxPC-3 and SW1990 pancreatic cancer cells, and similar to the qRT-PCR and western blotting results, PANC-1 and MIA PaCa-2 cells did not express substantial ASS protein *in situ* (Figure 1B). Furthermore, we analyzed the levels of ASS protein in 14 fresh-frozen pancreatic cancer tissue samples by western blotting and found that pancreatic cancers expressed low levels of the ASS protein (7 with ASS expression deficiency) (Figure 1D). Nine of fourteen tissue specimens were extracted for detection of ASS mRNA level, and the results show that transcriptional levels of the ASS gene were similar to its protein expression in the examined specimens (Figure 1E). Additionally, the expression of p65 (a subunit of heterodimeric NF-κB complexes) and caspase-3 (a proapoptotic protein) was evaluated in 14 pancreatic cancer tissue samples by western blotting, presenting that there was a constitutional expression of p65 and caspase-3 proteins in examined specimens, and high level of caspase-3 expression was associated with low p65 expression (*r* = −0.634, *P* = 0.027; Figure 1F).

### Expression of ASS is associated with unfavorable biological behaviours in pancreatic cancer

To understand the clinical importance of ASS expression in primary human pancreatic cancer tissues, we evaluated ASS expression in human pancreatic cancer tissues by IHC method. ASS expression was detected in 19 of the 34 (56%) specimens and results from 2 of those tissues are shown (Figure 1G, i-iv). Reduced ASS expression correlated with lymph node metastasis, and local invasion in patients with pancreatic cancer (Table 1). In addition, the expression of survivin, a member of the inhibitor of apoptosis protein (IAP) family, was also detected in the same cancer specimens, and its expression was found in cytoplasm and/or nucleus in most of the cancer specimens (25/34, 74%) (Figure 1G, v and vi). By comparing the expression of ASS and survivin in pancreatic cancer specimens, a positive correlation between reduced survivin and ASS expression was exhibited (Table 2).

**Table 1:** **Relation between clinical and histological characteristics of pancreatic cancer patients and ASS expression**

Characteristics	ASS	
	Positive	Negative
Median age (range) years	63 (47–78)	68 (44–83)
Sex (male:female)	11:8	10:6
Histological grade		
I	3 (9%)	2 (6%)
II	12 (35%)	9 (26%)
III	4 (12%)	4 (12%)
Tumor size (cm)		
≤ 3	6 (18%)	3 (9%)
> 3– ≤ 6	10 (29%)	11 (32%)
>6	3 (9%)	1 (3%)
Pathologic stage		
I	3 (9%)	1 (3%)
II	11 (32%)	8 (24%)
III	2 (6%)	2 (6%)
IV	3 (9%)	4 (12%)
Lymph node metastasis		
Positive	9 (26%)	13 (35%)
Negative	10 (29%)	2 (9%)*
Local invasion		
Positive	7 (24%)	12 (29%)
Negative	12 (32%)	3 (15%)*

*P < 0.05.

**Table 2:** **Correlation between survivin expression and ASS expression**

		ASS	
		Positive	Negative
Survivin	Positive	11 (41%)	14 (41%)
	Negative	8 (15%)	1 (3%)*

*P < 0.05.

### Effect of ADI on the growth, apoptosis, and cell cycle of pancreatic cancer cells

**Figure 2:**
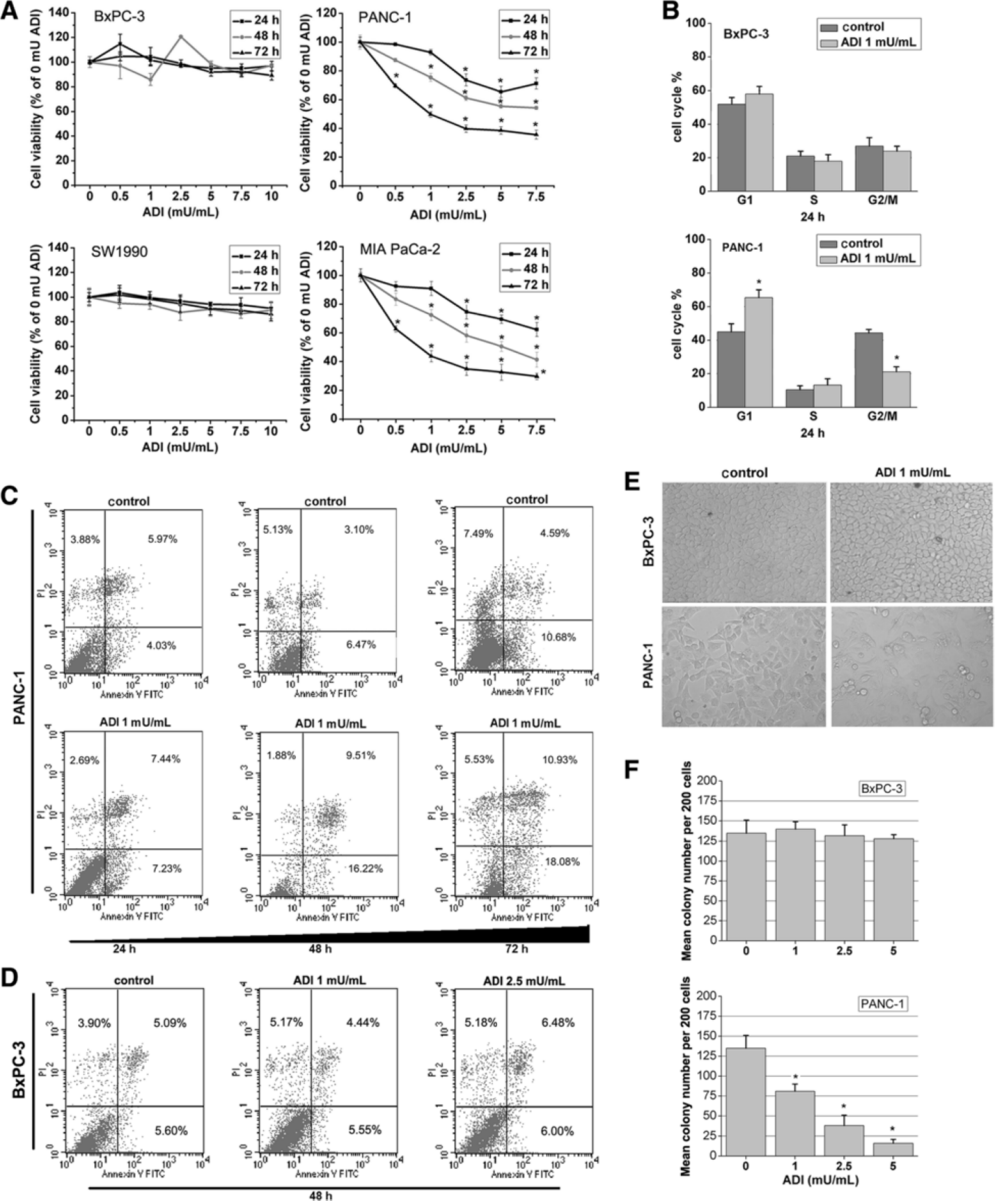
**The effect of ADI on the cell proliferation, apoptosis, cell cycle, and colony formation of pancreatic cancer cells. A**, The proliferation-inhibitory effect of ADI on BxPC-3, PANC-1, SW1990, and MIA PaCa-2 cells was measured by the MTT assay. *, *P* < 0.05 as compared with the control group (0 mU/mL ADI). **B**, Cell cycle progression of primary pancreatic cancer cell lines BxPC-3 and PANC-1 after treatment with or without ADI was analyzed by FACS. *, *P* < 0.05 as compared with the control group (0 mU ADI/mL); NS, not significant. **C**, The percentage of apoptotic PANC-1 cells treated with ADI was calculated by FACS. **D**, The percentage of apoptotic BxPC-3 cells treated by ADI for 48 h was calculated by FACS. **E**, ADI (1 mU/mL) intervention altered cell morphology of PANC-1 cells but not BxPC-3 cells. **F**, The colony forming ability of PANC-1 cells was altered by ADI intervention, while BxPC-3 colony formation was not changed. *, *P* < 0.05 as compared with the control group (0 mU ADI/mL).

Next, we examined the cytotoxic effect of ADI on BxPC-3, SW1990, MIA PaCa-2, and PANC-1 cells by the MTT assay. Following treatment for one to three days, ADI significantly decreased the viability of ASS-deficient PANC-1 and MIA PaCa-2 cells in a dose- and time- dependent manner, but did not inhibit the proliferation of ASS-expressing BxPC-3 and SW1990 (Figure 2A). The 50% inhibitory concentration (IC_50_) of ADI in PANC-1 was determined to be 1 mU/mL at 72 h and was used as the treatment dose in ensuing experiments. Subsequently, primary pancreatic cancer cell lines PANC-1 and BxPC-3 were used in further cellular and molecular experiments. The two cell lines treated with ADI or PBS were analyzed for cell cycle progression and apoptosis using FACS analysis. The findings showed that 1 mU/mL of ADI induced PANC-1 cell cycle arrest at the G1 phase and with a shorter G2/M phase, but caused scarcely any delay at the respective cell cycle phase for the BxPC-3 cell line at 24 h (Figure 2B). Similarly, 1 mU/mL ADI induced significant programmed cell death in ASS-deficient PANC-1 cells at 24, 48, and 72 h following treatment (Figure 2C), but did not cause apoptosis in ASS-positive BxPC-3 cells at 48 h (Figure 2D). In addition, the cellular morphology of PANC-1 cells was altered (Figure 2E) and the colony formation ability was attenuated upon treatment with 1 mU/mL of ADI (Figure 2F), but these cellular changes were not observed in BxPC-3 cells.

### Regulatory role of ADI on the expression of apoptosis-related proteins, cell cycle protein cyclin D1, and phosphorylation of STAT3, AKT, and NF-κB p65

**Figure 3:**
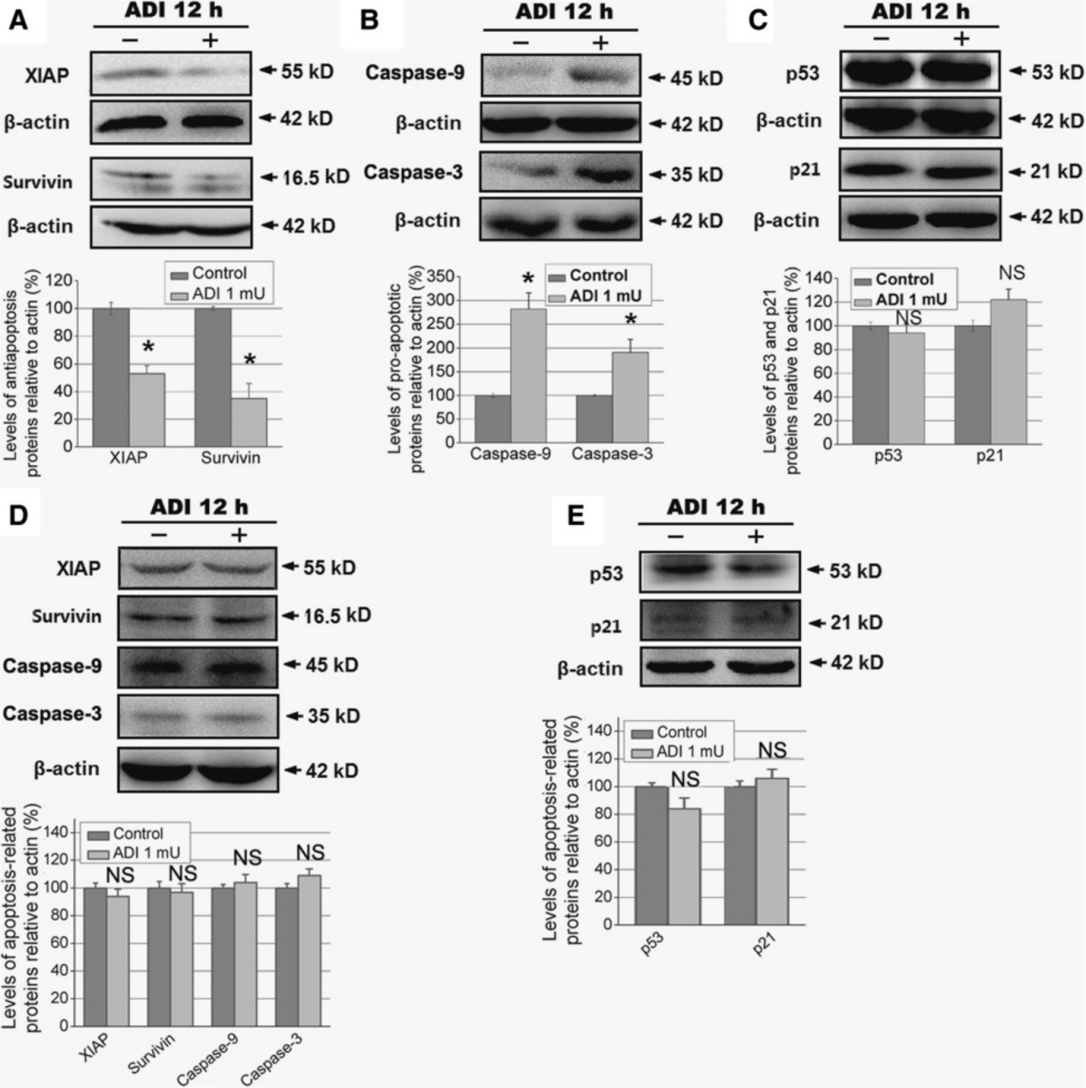
**The effect of ADI on apoptosis-related proteins and cell cycle protein cyclin D1 in pancreatic cancer cells. A** and **B**, Treatment with ADI (1 mU/mL) regulates the levels of antiapoptotic proteins XIAP and survivin, and pro-apoptotic proteins caspase-3 and caspase-9 in PANC-1 cells. *, *P* < 0.05 as compared with the control group (0 mU/mL ADI); NS, not significant. **C**, ADI does not alter the expression level of p53 and p21 proteins in PANC-1 cells, as compared with the control group. **D** and **E**, ADI does not alter the expression level of p53 and p21 proteins in BxPC-3 cells, as compared with the control group.

**Figure 4:**
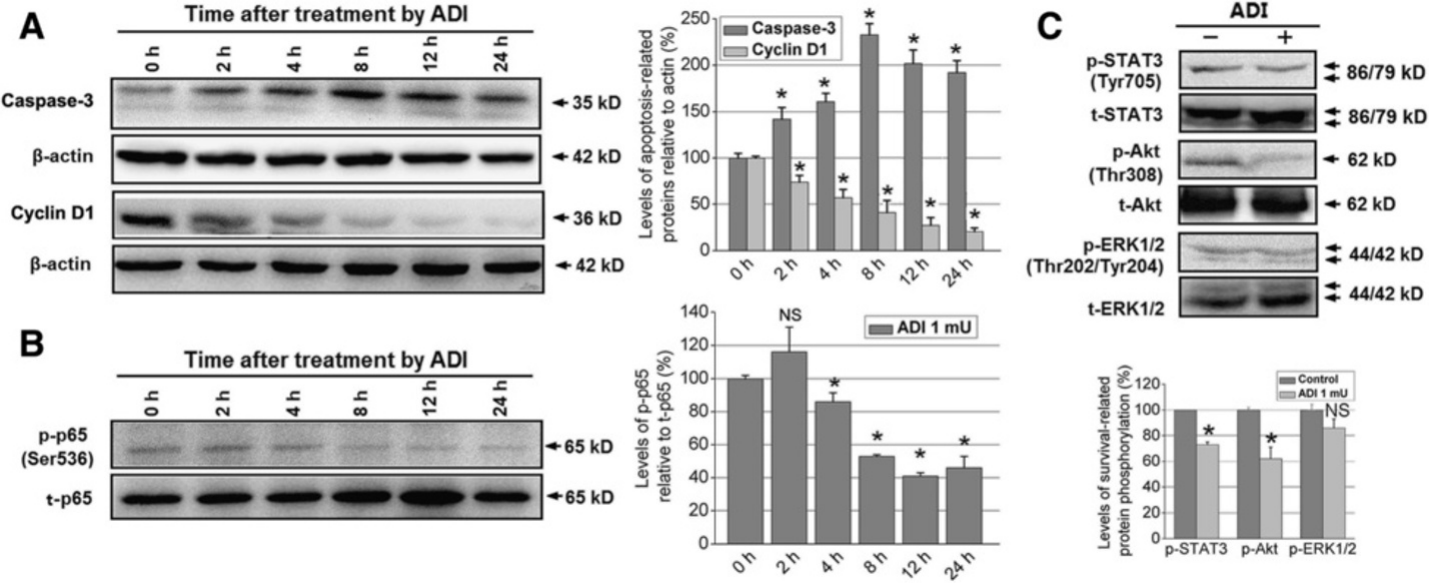
**The effect of ADI on caspase-3 and cyclin D1, and the phosphorylation of NF-κB p65, STAT3, Akt, and ERK1/2 in ASS-deficient PANC-1 cells. A**, ADI (1 mU/mL) up-regulates caspase-3 protein and decreases cell cycle protein cyclin D1 in a time-dependent manner in PANC-1 cells. *, *P* < 0.05 as compared with the treatment at 0 h; NS, not significant. **B**, ADI treatment (1 mU/mL for 0–24 h) of PANC-1 cells resulted in reduced phosphorylation of the NF-κB p65 subunit at Ser536. *, *P* < 0.05 as compared with the treatment group at 0 h; NS, not significant. **C**, The effect of ADI (1 mU/mL) on the phosphorylation of cell survival- associated proteins STAT3, Akt, and ERK1/2. *, *P* < 0.05 as compared with the control group; NS, not significant.

To explore the precise mechanisms of ADI-induced apoptosis in pancreatic cancer cells, we studied several apoptosis-related proteins using western blotting. Our findings showed that, after 12 h treatment, ADI significantly downregulated the expression of two IAP-family antiapoptotic proteins, namely X-linked IAP (XIAP) and survivin (Figure 3A), and simultaneously upregulated the expression of caspase-3 and caspase-9 that are responsible for the release of mitochondrial proapoptotic proteins (Figure 3B) in PANC-1 cells; however, the same concentration of ADI treatment did not alter the expression of these apoptosis-related proteins in BxPC-3 cells (Figure 3D). Next, we found considerable accumulation of p53 protein in the p53-mutant PANC-1 (Figure 3C) and BxPC-3 (Figure 3E) cells, but p53 expression was not significantly altered after ADI treatment in either cell line, and no significant change in p21 protein (a p53 induced product) expression was detected. Due to no significant cellular and molecular changes in ASS-positive BxPC-3 pancreatic cancer cell after ADI treatment, we focused on the relevant studies in the ASS-negative PANC-1 cell line. After 0 to 24 h treatment with ADI, caspase-3 activation increased progressively in a time-dependent fashion, while the expression of cyclin D1 was reduced in PANC-1 cells (Figure 4A). Furthermore, we tested the phosphorylation levels of p65 at serine 536 (p-p65 [Ser536]), shown to play a critical role in the activation of the NF-κB pathway [39, 40], in PANC-1 cells treated with ADI at several time points. We discovered that p-p65 (Ser536) decreased in a time-dependent manner following ADI treatment (Figure 4B). To understand whether ADI treatment blocked the phosphorylation of NF-κB p65 in PANC-1 cells via altering survival signaling, we detected the levels of Akt, p-Akt, ERK1/2, p-ERK1/2, STAT3, and p-STAT3. The results showed that ADI treatment for 8 h inhibited phosphorylation of STAT3 and Akt, but not ERK1/2 (Figure 4C).

### Effect of ADI on GEM-induced cytotoxicity in pancreatic cancer cells

**Figure 5:**
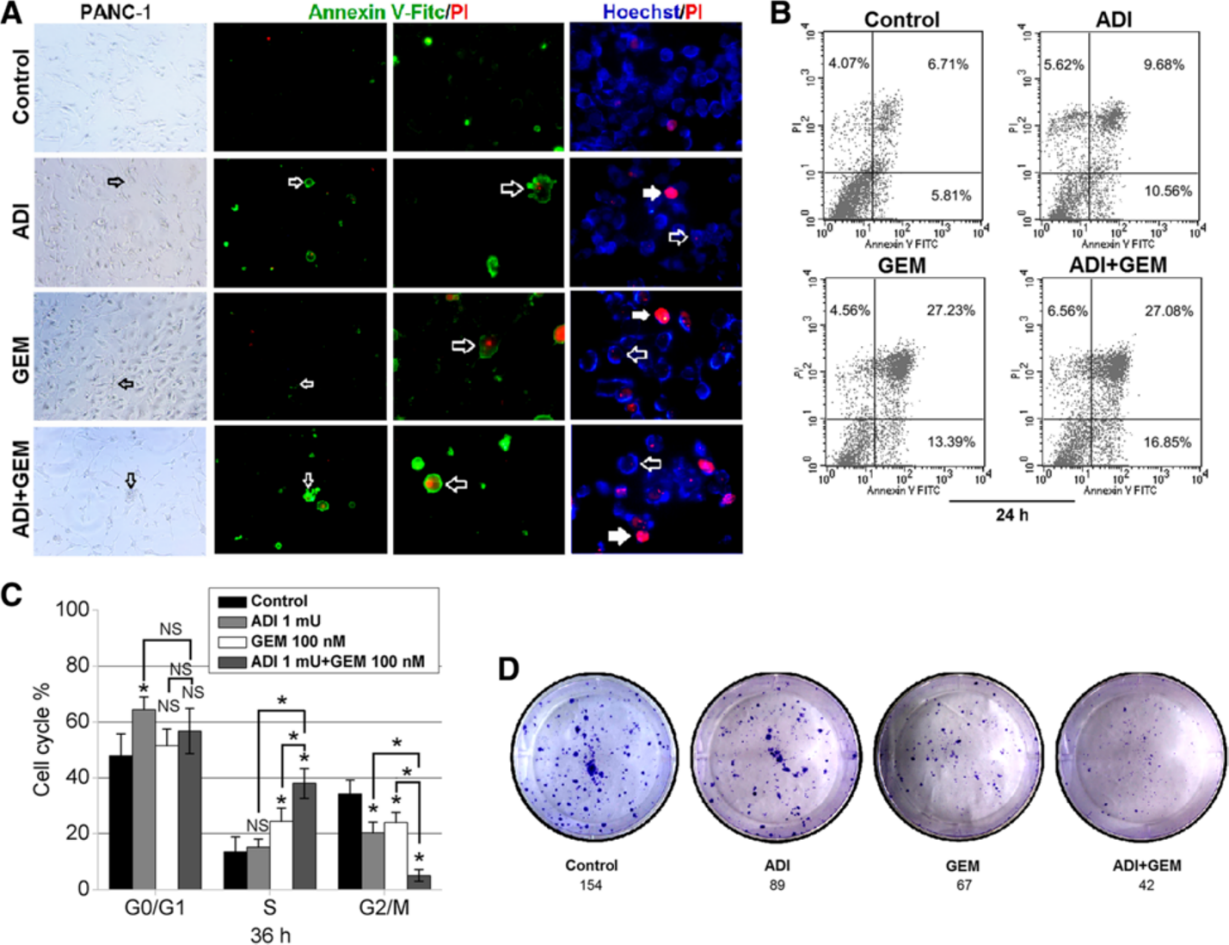
**The impact of ADI combined with GEM on PANC-1 cell apoptosis, cell cycle arrest, and inhibition of colony formation. A**, Apoptosis in PANC-1 cells at the 24 hour time point in the control group, ADI alone, GEM alone, or both ADI and GEM groups was visualized by *in situ* fluorescence microscopy using the indicated dyes (Annexin V-FITC/PI or Hoechst 33258/PI). **B**, The percentages of apoptotic PANC-1 cells treated with vehicle, ADI alone, GEM alone, or both drugs were estimated by FACS analysis following staining with Annexin V-FITC/PI. **C**, Cell cycle progression of PANC-1 cells following treatment with ADI or GEM alone or in combination was analyzed by single staining with PI. *, *P* < 0.05, as compared with the control group or indicated groups. **D**, Proliferative activity of PANC-1 cells treated by GEM following ADI was viewed by the single cell colony formation assay.

To evaluate the antiproliferative activity of ADI that potentiated GEM treatment, the MTT assay was initially conducted. The IC_50_ of GEM that inhibited the proliferation of BxPC-3 (Additional file 2: Figure S2A) and PANC-1 (Additional file 2: Figure S2B) cells was estimated by the MTT assay to be approximately 30 nM and 100 nM at 72 h, respectively; this concentration was then used in the subsequent experiments. GEM in combined with 6 h ADI pretreatment significantly inhibited the proliferation of PANC-1 cells compared to ADI or GEM alone (Additional file 2: Figure S2D), but this effect was not observed in BxPC-3 cells (Additional file 2: Figure S2C). Furthermore, by using *in situ* fluorescence microscopy visualization (Figure 5A) and FACS (Figure 5B), it was revealed that ADI pretreatment for 6 h promoted GEM-induced PANC-1 cell apoptosis by 24 h. We found that the apoptotic cells *in situ* readily stained with Annexin V-FITC/PI (green and red fluorescence) as well as with Hoechst and PI (blue and red fluorescence) (Figure 5A). Additionally, the GEM mediated S phase-arrest was enhanced in the case of pretreatment with ADI for 6 h (Figure 5C). We conducted a colony formation assay to test the colony-formation potential of individual PANC-1 cells; the results showed that GEM in combination with ADI pretreatment for 6 h reduced the colony numbers of PANC-1 cells compared to GEM or ADI alone (Figure 5D).

### Effect of ADI on the transcription levels of apoptosis-related genes

**Figure 6:**
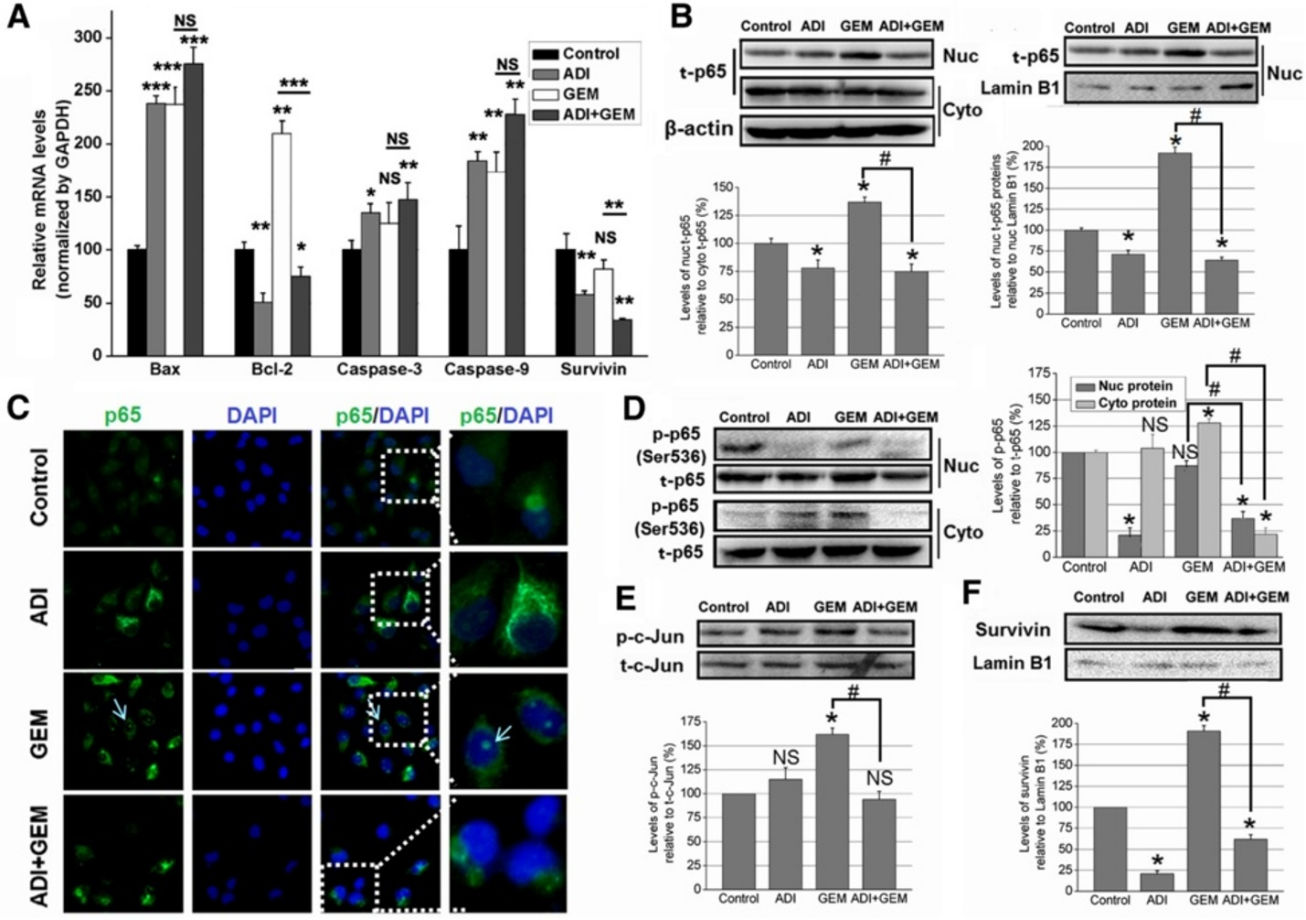
**The molecular changes on ADI treatment enhanced the sensitivity of PANC-1 cells to GEM. A**, The mRNA levels of Bax, Bcl-2, caspase-3, and -9, and survivin were examined in four experimental groups of PANC-1 cells by qRT-PCR analyses. *, *P* < 0.05, as compared with the control group or indicated groups; NS, not significant. **B**, The ratio of nuclear p65 to cytoplasmic p65 protein expression, and the relative expression of p65 protein in nuclear extracts were tested in control, ADI, GEM, or ADI + GEM-treated PANC-1 cells. *, *P* < 0.05, as compared with the control group; ^#^, *P* < 0.05, as compared with the indicated groups; NS, not significant. **C**, Inhibition of GEM-induced p65 nuclear translocation by ADI in PANC-1 cells was visualized by *in situ* immunofluorescence microscopy. **D**, The ratio of p-p65 (Ser536) to total (t)-p65 protein expression in nuclear and cytoplasmic extracts was tested in PANC-1 cells treated with experimental treatments. *, *P* < 0.05, as compared with the control group; ^#^, *P* < 0.05, as compared with the indicated groups; NS, not significant. **E**, The phosphorylation levels of c-Jun protein (Ser73) in nuclear extracts of PANC-1 cells treated with various agents were detected by western blotting. *, *P* < 0.05, as compared with the control group; ^#^, *P* < 0.05, as compared with the indicated groups; NS, not significant. **F**, The levels of IAP survivin protein in nuclear extracts from treated-PANC-1 cells were detected by western blotting. *, *P* < 0.05, as compared with the control group; ^#^, *P* < 0.05, as compared with the indicated groups.

To further understand the molecular changes associated with ADI-mediated potentiation of GEM-induced apoptosis in PANC-1 cells, we examined the mRNA levels of Bax, Bcl-2, caspase-3, and -9, and survivin in the cells by qRT-PCR. As shown in Figure 6A, both ADI and GEM upregulated Bax, caspase-3 and -9 mRNA levels; GEM induced the mRNA level of the antiapoptotic gene Bcl-2; while ADI not only decreased the expression of Bcl-2 but also inhibited the induction of Bcl-2 transcription by GEM (with ADI pretreatment for 6 h). Additionally, ADI downregulated survivin mRNA, while GEM had limited impact on survivin gene transcription; however, the combination of ADI pretreatment for 6 h with GEM resulted in even greater inhibition of survivin expression than ADI alone.

### ADI suppresses phosphorylation (serine 536) and nuclear translocation of NF-κB p65 protein

We next determined whether ADI potentiated the GEM-induced apoptosis of PANC-1 cells by blocking activation of the NF-κB pathway. We found that ADI pretreatment for 6 h downregulated the nuclear expression of p65 and inhibited p65 induction by GEM (Figure 6B). Next, we studied whether ADI inhibited the GEM-induced p65 nuclear translocation in PANC-1 cells using *in situ* immunofluorescence microscopy. As shown in Figure 6C, p65 was located in the cytoplasm in the control group and in the ADI-treated group, while a significant amount of p65 was visible in the nucleus as green fluorescent spots in the GEM-treated group. However, when PANC-1 cells treated with both GEM and the pretreatment ADI for 6 h, the green fluorescent nuclear spots of p65 disappeared, indicating that ADI can block the nuclear translocation of the NF-κB p65 subunit. To evaluate whether ADI regulates GEM-induced activation of NF-κB pathway by inhibiting p65 phosphorylation, we examined the ratio of p-p65 (Ser536) to total p65 in nuclear and cytoplasmic extracts. Our analysis of the nuclear proteins showed that ADI significantly decreased p-p65 expression levels, while GEM did not. Additionally, ADI pretreatment reduced p-p65 levels in combination with GEM. In the cytoplasmic extracts, GEM significantly increased p-p65 expression which was unaffected by ADI alone, but significantly reduced in ADI pretreatment combined with GEM (Figure 6D). Together, these data provide evidence that ADI can suppress NF-κB pathway activation. Furthermore, GEM alone induced c-Jun phosphorylation at Ser73, but ADI alone or together with GEM did not. However, ADI pretreatment for 6 h could decrease the p-c-Jun induction by GEM alone, indicating that ADI could maintain c-Jun phosphorylation at the baseline level (Figure 6E). Finally, we validated the expression of survivin protein in nuclear extracts and found results similar to that for mRNA expression inhibited by ADI treatment; however, GEM increased the expression of nuclear survivin (Figure 6F).

### ADI blocks NF-κB p65 phosphorylation (serine 536) via inactivating PI3K/Akt survival signal pathway

**Figure 7:**
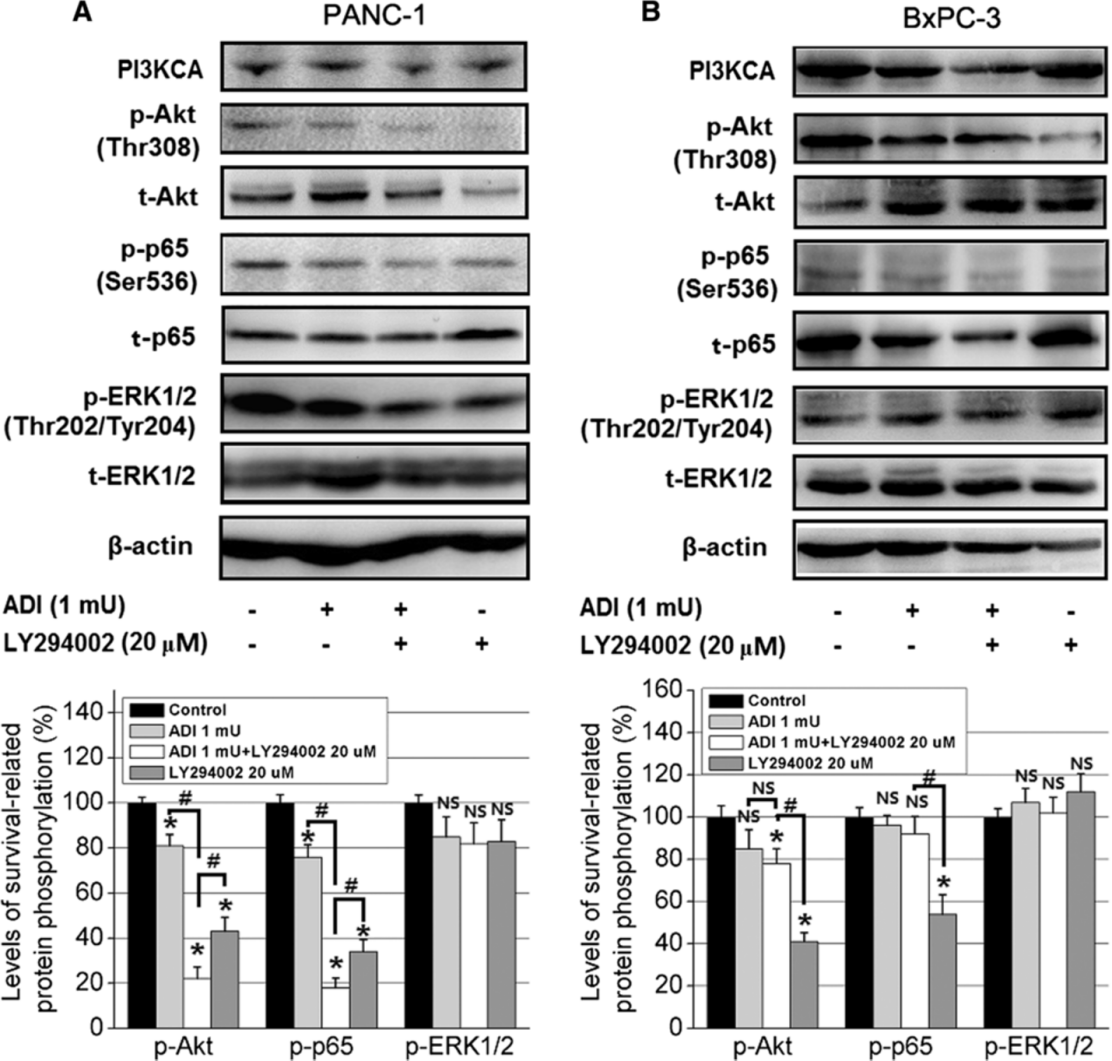
**The effect of ADI in combination with PI3K inhibitor LY294002 on PI3K/Akt survival signal pathway.** Protein expression levels of phosphorylated and total levels of Akt, p65, Erk1/2 along with PI3KCA and B-actin were detected in **A**, PANC-1 cells and **B,** BxPC-3 cells treated with or without ADI and LY294002. The combined treatment significantly reduced p-Akt and p-p65 expression in PANC-1, but not BxPC-3 cells, as shown in the densitometry graphs. *, *P* < 0.05, as compared with the control group; ^#^, *P* < 0.05, as compared with the indicated groups; NS, not significant.

To understanding whether ADI down-regulated the phosphorylation of NF-κB p65 by blocking activation of the PI3K/Akt survival signal pathway, we evaluated the expression of several important proteins in this signaling pathway in pancreatic cancer cells treated with ADI in combination with the PI3K inhibitor LY294002 treatment for 20 min. The results showed that ADI combined with LY294002 at 20 μM significantly down-regulated the level of p-Akt (Thr308) and p-p65 (Ser536), but not p-ERK1/2 (Thr202/Tyr204) in ASS-deficient PANC-1 cells, as compared to ADI treatment alone (*P* < 0.05) (Figure 7A); however, the combined treatment of ADI and LY294002 did not change the expression of relevant proteins of the PI3K/Akt and NF-κB p65 signaling pathways as compared with ADI treatment alone, in ASS-positive BxPC-3 pancreatic cancer cells (Figure 7B).

### ADI augments GEM-mediated inhibition of tumorigenesis of PANC-1 pancreatic cancer cells *in vivo*

**Figure 8:**
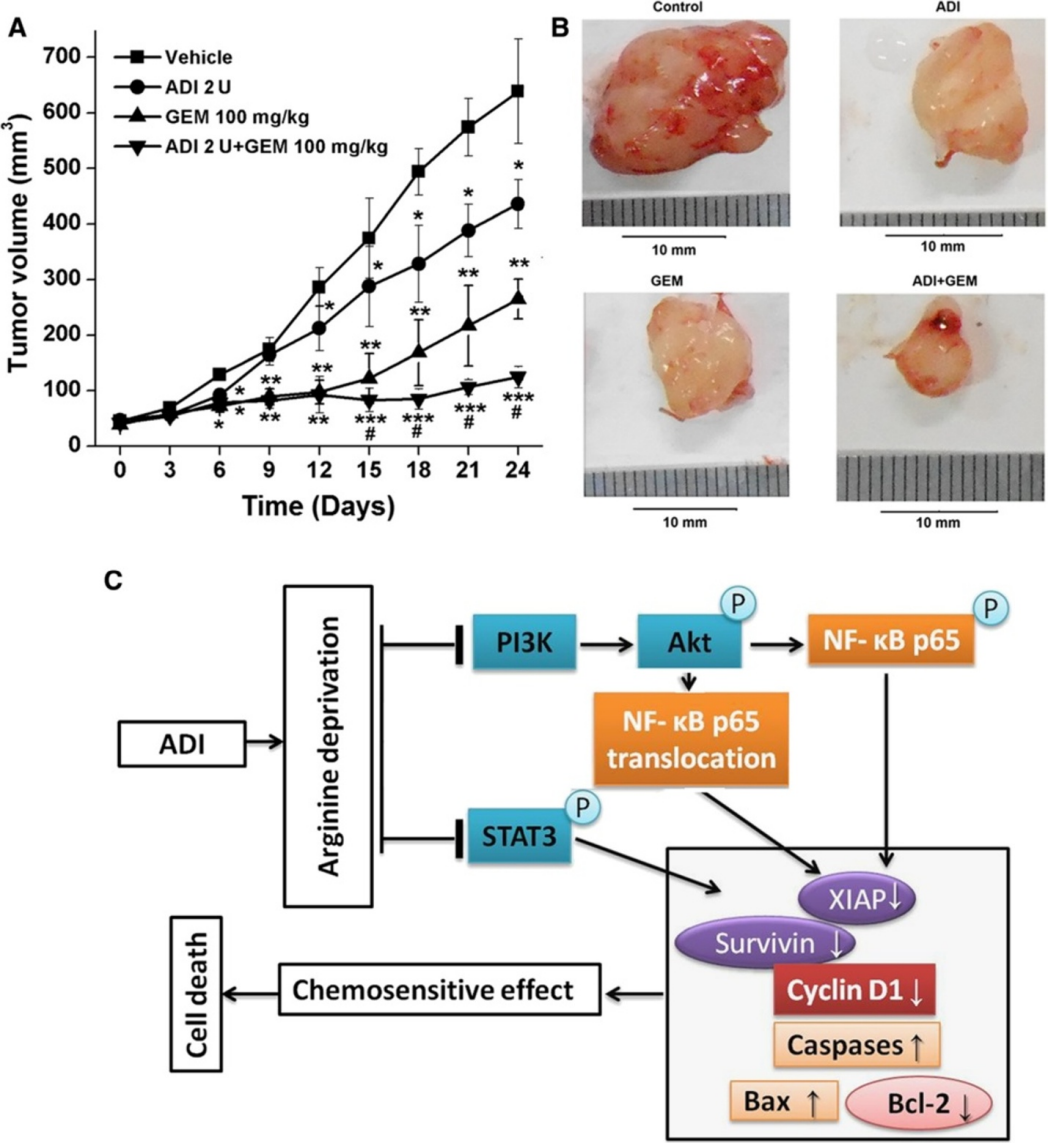
**ADI promoted chemosensitivity to GEM** ***in vivo*** **. A**, The effect of ADI and/or GEM treatment on the growth of PANC-1 tumors in BALB/c athymic mice are shown. All treatment groups resulted in significant reduction in tumor volume after 24 days relative to the control group; however, the treatment with both ADI and GEM resulted in the greatest growth inhibition overall. Further, ADI + GEM significantly inhibited tumor growth when compared to GEM alone during days 15 to day 24. *, *P* < 0.05, **, *P* < 0.01, ***, *P* < 0.001 as compared with vehicle group. ^#^, *P* < 0.05, as compared with GEM or ADI alone group. **B**, Representative images of pancreatic tumors obtained from the mice treated with control, ADI, GEM, or ADI + GEM are shown. The tumors were collected at the end of the 24-day treatment period. The combined treatment of ADI + GEM resulted in the greatest reduction of tumor growth. **C**, Proposed model for how ADI sensitizes pancreatic cancer cells to GEM and results in cell death. ADI deprives cells of arginine which leads to inhibition of STAT and PI3K/Akt pathways. As a consequence, NF-κB activity and nuclear translocation are reduced leading to decreased synthesis of pro-survival proteins and increased synthesis of pro-death proteins. These events sensitize pancreatic cancer cells to chemotherapy (GEM) resulting in cell death.

Based on our findings that ADI blocks NF-κB signaling, leading to enhance GEM-induced apoptosis of PANC-1 cells *in vitro*, we thus sought that it would be interesting to determine if ADI enhanced the antitumor effect of GEM *in vivo*. For these studies, PANC-1 cells were subcutaneouly implanted into nude mice to generate a xenograft. When the xenograft tumors had grown to approximately 50 mm^3^, the mice (n = 6) were treated with PBS (vehicle), ADI (2 U/mouse), GEM (100 mg/kg), or both ADI and GEM. The tumors regressed during the treatment period in all groups except the vehicle group (Figure 8A and B). From days 15–24, treatment with both ADI and GEM markedly regressed tumor growth when compared to GEM or ADI alone, and minimal tumors were observed in the combination group at resection (Figure 8B).

## Discussion

In this report, we show that reduced ASS expression is correlated with unfavorable tumor behaviors in patients with pancreatic cancer, and arginine deprivation by ADI can effectively induce programmed cell death in PANC-1 cells with undetectable ASS expression, and also sensitize pancreatic cancer cells to GEM, a first-line chemotherapy in pancreatic cancer. In addition, we demonstrate the mechanism by which ADI augments the sensitivity of ASS-deficient pancreatic cancer cells to GEM treatment. Our findings show that ADI alone results in down-regulation of IAP family member survivin and XIAP, and induces caspase-dependent apoptosis. Furthermore, ADI may block PI3K/Akt and STAT3 survival signaling to exhibit antitumor effects. By blocking PI3K/Akt signaling and suppressing NF-κB activation via inhibition of the nuclear translocation and phosphorylation (serine 536) of nuclear NF-κB p65, ADI displays a highly significant synergism in anticancer activities against pancreatic cancer cells deficient in ASS expression in combination with GEM (Figure 8C). Thus, the present study offers a new treatment strategy for pancreatic cancer involving arginine depletion.

ADI has been reported to have antitumor activity, it is antiangiogenic, and it synergizes to promote dexamethasone-induced cytotoxicity [10, 14, 15, 41–43]. As described in previous studies cancer cells are sensitive to the antiproliferative activity of ADI arginine deprivation, which correlates with the endogenous arginine metabolic enzyme, ASS [10, 13, 15]. Here, we found that the majority of human pancreatic cancer specimens have a low expression of ASS, and reduced ASS expression was associated with unfavorable histopathological characteristics of pancreatic cancer. In four of the pancreatic cancer cell lines that were examined, the ASS mRNA levels by qRT-PCR assay were similar as previously reported, but expression in SW1990 was not detected (Figure 1A); as a pilot study on ADI apply to treatment for pancreatic cancer, this work demonstrated the effect of PEG-ADI on cell growth inhibition and apoptosis induction in MIA PaCa-2 pancreatic cancer cells [15]. However, the study was worthwhile to understand the precise mechanism of ADI-induced pancreatic cancer cell growth inhibition and apoptosis induction. The present study contributed to our understanding of some of the signaling mechanisms regulated by ADI in ASS-deficient PANC-1 cells. Naturally, our results are likely to be generally applicable to other pancreatic cancer cells that lack ASS expression, other than the cell lines used in this study.

A number of studies demonstrate that GEM treatment can induce NF-κB signal activation [23, 44]. NF-κB activation is involved in the inhibition of apoptosis, induction of mitogenic gene products such as cyclin D1, increased expression of proangiogenic factors, and regulation of gene products that promote migration and invasion of pancreatic cancer cells, which together contribute to the chemoresistance of pancreatic cancer [45–49]. By arginine depletion, ADI causes metabolic stress in arginine auxotrophic cells, which could compliment conventional GEM-based chemotherapies that are largely based on genotoxic stress. In our report, fourteen pancreatic cancer specimens had varying expression levels of NF-κB p65 and showed an inverse correlation with the expression of caspase-3. Our *in vitro* studies showed that ADI upregulates many proapoptotic factors, such as Bax, caspase-3 and −9, and simultaneously downregulates antiapoptotic gene products, Bcl-2, XIAP, and survivin, but not p53 and p21, indicating that ADI promoted apoptosis in PANC-1 cells in part via caspase activation and suppression of IAPs. ADI treatment blocked the phosphorylation of NF-κB p65 subunit. With the combination therapy of ADI and GEM, we found that ADI obstructs the GEM-mediated NF-κB p65 protein translocation into the nucleus where NF-κB binds with various genes and activates their transcription [50–52]. By suppressing p65 subunit nuclear translocation and phosphorylation at Ser536, the two NF-κB activation pathways [25, 26, 39, 40], ADI reduces chemoresistance, producing a synergism on with GEM-induced programmed cell death in PANC-1 pancreatic cancer cells. Moreover, a preliminary animal experiment also validated the synergism of ADI with the antitumor effect of GEM *in vivo*. Our study provides the first data suggesting that ADI enhances the chemosensitivity to GEM by suppression of NF-κB p65 nuclear translocation and phosphorylation of nuclear p65 subunit, and thus, the work described here offers a new treatment option for pancreatic cancer.

Multiple signals regulate or synergize with NF-κB pathway activation, such as PI3K/Akt [25, 26], STAT3 [45], and MAPK/ERK1/2 [53, 54]. We explored whether ADI alters the phosphorylation levels of cell survival-associated signaling pathway proteins, including Akt, STAT3, and ERK1/2. Our findings revealed that ADI reduced p-Akt and p-STAT3 levels, but not p-ERK1/2. Based on our experiments with ADI treatment alone, we postulate that ADI may obstruct nuclear translocation of NF-κB p65 subunit and downregulate nuclear p65 phosphorylation at Ser536 via suppression of PI3K/Akt signaling under metabolic stress by arginine depletion. Indeed, the molecular studies in pancreatic cancer cells verified this hypothesis.

## Conclusion

Although GEM-based chemotherapies are currently the standard of care for the treatment of advanced pancreatic cancer, its efficacy is limited because pancreatic cancer cells are often resistant to GEM through a mechanism that involves NF-κB activation. In this context, our findings suggest that reduced ASS expression predicts unfavorable tumor behaviours, while in ASS-deficient PANC-1 cells, ADI enhances their chemosensitivity to GEM-induced apoptosis. The mechanism by which ADI synergized with GEM involved inhibiting PI3K/Akt/NF-κB signaling, as evidenced by NF-κB pathway inactivation via the suppression of nuclear translocation and phosphorylation of the p65 subunit at Ser536. Therefore, the combination of ADI and GEM is advantageous because of their complementary action, and will offer a novel treatment strategy for pancreatic cancer.

## Electronic supplementary material

Additional file 1: Figure S1: The proteinogram of heat-denatured ADI protein from *M. arginini*. The ADI gene was cloned from *M. arginini* genomic DNA and recombinant ADI was overexpressed and purified as previously described [31]. The molecular weight of purified ADI was observed to be 46 kDa. (TIFF 1 MB)

Additional file 2: Figure S2: ADI sensitizes pancreatic cancer cells to GEM-induced growth inhibition. **A**, GEM inhibited the proliferation of BxPC-3 cells at continuous concentrations estimated by the MTT growth assay. **B**, GEM inhibited the proliferation of PANC-1 cell growth estimated by the MTT growth assay. **C**, ADI in combination with GEM did not increase the inhibition ratio of proliferation in BxPC-3 cells more than that of treatment with GEM alone. **D**, ADI in combination with GEM significantly increased the inhibition ratio of proliferation in PANC-1 cells compared to treatment with ADI or GEM alone. * *P* < 0.05, ** *P* < 0.01, *** *P* < 0.001 as compared with control group or indicated groups. (TIFF 7 MB)

## Abbreviations

ADI: Arginine deiminase
Akt: Protein kinase B
ASS: Argininosuccinate synthetase
ATCC: American Type Culture Collection
BCA: Bicinchoninic acid
c-Jun: A member of activating protein 1 family
DAB: 3,3-diaminobenzidine tetrahydrochloride
DAPI: Diamidino-2-phenylindole
DMSO: Dimethylsulfoxide
ERK1/2: Extracellular signal-regulated kinases 1 and 2
GAPDH: Glyceraldehyde-3-phosphate dehydrogenase
GEM: Gemcitabine
HCC: Hepatocellular carcinoma
IAP: Inhibitor of apoptosis protein
IHC: Immunohistochemical
MAPK: Mitogen-activated protein kinase
MTT: Methyl thiazolyl tetrazolium
NF-κB: Nuclear factor-κB
PI: Propidium iodide
PI3K: Phosphatidylinositol-3-kinase
qRT-PCR: Quantitative-real time reverse transcription polymerase chain reaction
Ser: Serine
STAT3: Signal transducer and activator of transcription 3
Thr: Threonine
Tyr: Tyrosine
XIAP: X-linked IAP.
