# Supplementary figures and images for: Arginine deiminase augments the chemosensitivity of argininosuccinate synthetase-deficient pancreatic cancer cells to gemcitabine via inhibition of NF-κB signaling
Source: BMC Cancer. 2014 Sep 20;14:686. doi: 10.1186/1471-2407-14-686 (PMC4189535; doi:10.1186/1471-2407-14-686)

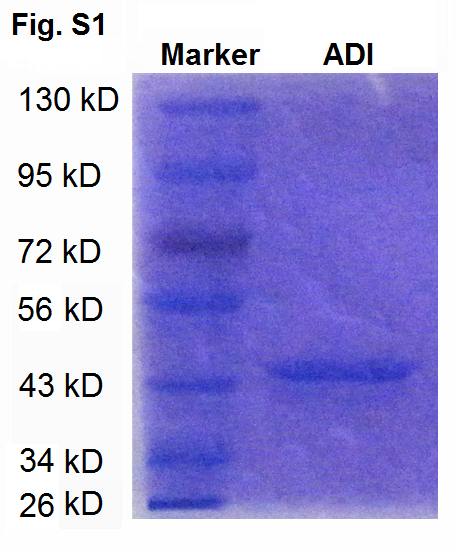

Supplement: Supplementary file 1 — Additional file 1: Figure S1: The proteinogram of heat-denatured ADI protein from M. arginini. The ADI gene was cloned from M. arginini genomic DNA and recombinant ADI was overexpressed and purified as previously described [31]. The molecular weight of purified ADI was observed to be 46 kDa. (TIFF 1 MB) [file 12885_2014_4882_MOESM1_ESM.tiff]

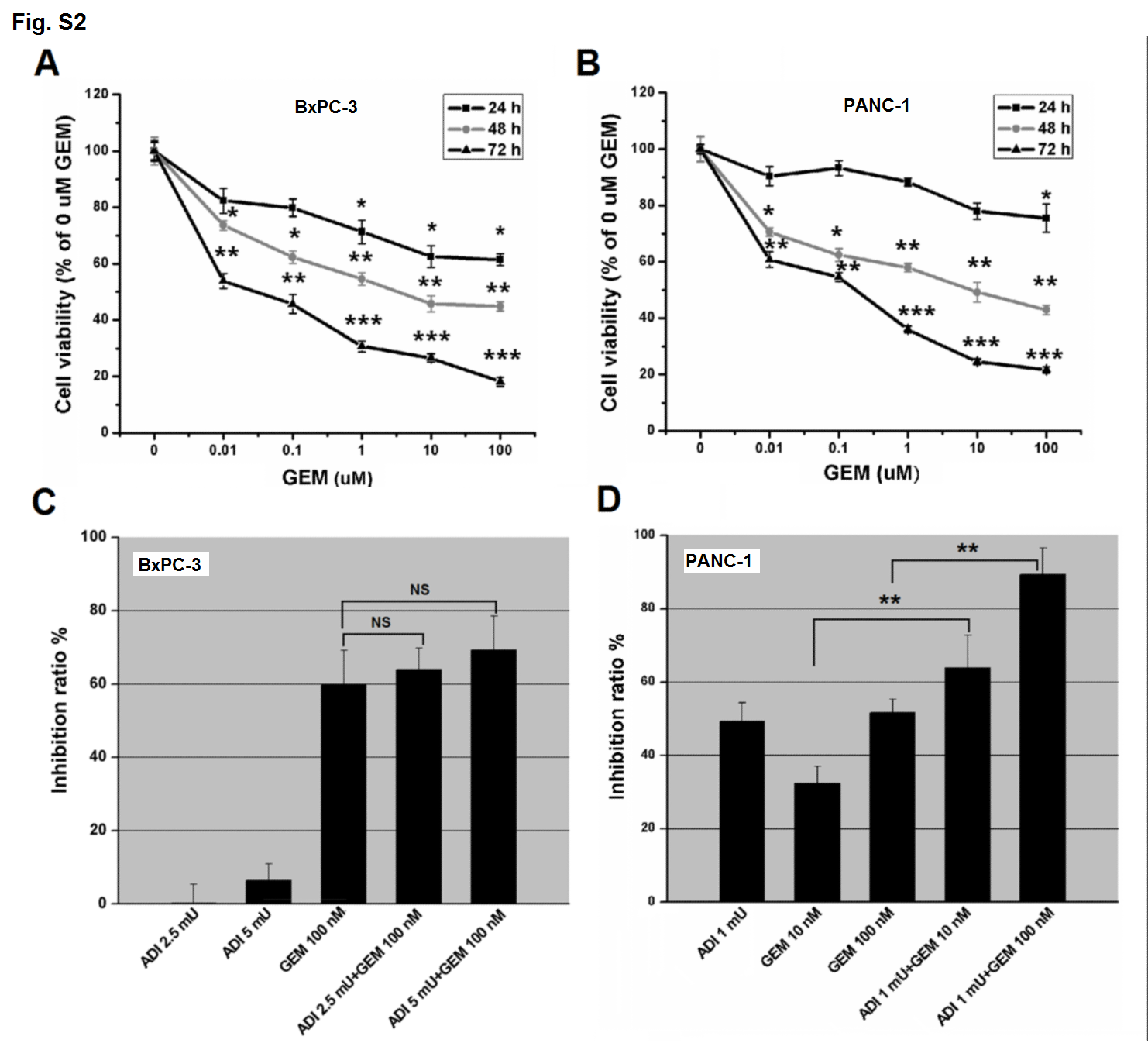

Supplement: Supplementary file 2 — Additional file 2: Figure S2: ADI sensitizes pancreatic cancer cells to GEM-induced growth inhibition. A, GEM inhibited the proliferation of BxPC-3 cells at continuous concentrations estimated by the MTT growth assay. B, GEM inhibited the proliferation of PANC-1 cell growth estimated by the MTT growth assay. C, ADI in combination with GEM did not increase the inhibition ratio of proliferation in BxPC-3 cells more than that of treatment with GEM alone. D, ADI in combination with GEM significantly increased the inhibition ratio of proliferation in PANC-1 cells compared to treatment with ADI or GEM alone. * P < 0.05, ** P < 0.01, *** P < 0.001 as compared with control group or indicated groups. (TIFF 7 MB) [file 12885_2014_4882_MOESM2_ESM.tiff]
